# Supplementary material for: Active topological glass
Source: Nat Commun. 2020 Jan 7;11:26. doi: 10.1038/s41467-019-13696-z (PMC6946665; doi:10.1038/s41467-019-13696-z)
Supplement: Supplementary file 1 — Supplementary Information [file 41467_2019_13696_MOESM1_ESM.pdf]

Supplementary Information: Active topological glass

Jan Smrek,[1,2] Iurii Chubak,[1] Christos N. Likos,[1] and Kurt Kremer[2]
[1]Faculty of Physics, University of Vienna, Boltzmanngasse 5, A-1090 Vienna, Austria
[2]Max Planck Institute for Polymer Research, Ackermannweg 10, 55128 Mainz, Germany

CONTENTS

Supplementary Note 1: Details on rings static and dynamic properties 1
Details on threadings 2
Supplementary Note 2: Emergent directionality of partly active polymers 6
Supplementary Note 3: Effective equilibrium model and single ring conformations 6
Supplementary Note 4: Additional properties of the steady state 7
Supplementary Note 5: Details on cut rings 9
Supplementary Note 6: Varying number of partly active chains 9
Supplementary Note 7: Varying ring length 9
Supplementary References 15

Supplementary Note 1: Details on rings static and dynamic properties

In Supplementary Figure 1, we present additional shape parameters of the rings, such as the asphericity a and the contact probability $P(s)$, as a function of time measured from the onset of activity.

After switching on the activity, the overall size of the rings grows, as indicated in Supplementary Figure 1**a** and Figure 1**b**, being a consequence of the directional dynamics of the rings in the presence of other chains. Simultaneously, the rings become more ashperical, as can be seen in Supplementary Figure 1**b** showing the dimensionless asphericity parameter a, defined as

$$a = \frac{\langle \lambda_1 - \frac{1}{2}(\lambda_2 + \lambda_3) \rangle}{\langle \lambda_1 + \lambda_2 + \lambda_3 \rangle} \qquad (1)$$

where λ_i's are the eigenvalues of the ring's gyration tensor $G_{ij} = \frac{1}{N}\sum_{n=1}^{N}(r_i^{(n)} - R_i)(r_j^{(n)} - R_j)$ ($r_i^{(n)}$ is the i-th component of the position vector $\mathbf{r}^{(n)}$ of the n-th particle and $\mathbf{R}$ is the center of mass position of the ring), ordered as $\lambda_1 \geq \lambda_2 \geq \lambda_3$. Furthermore, we checked that all rings are smaller than the linear box size at all times (see Supplementary Figure 1**c**). This is to make sure that the rings do not wrap around the periodic boundary conditions, which would cause unphysical dynamics due to self-threadings.

$P(s)$ represents the probability that two monomers separated by a contour distance s are in contact in 3D space. It is computed for each segment length s as the fraction of times the segments endpoints are within distance 1.5σ averaged over the segments position within the rings and averaged over rings. It becomes non-monotonic at short distances, as seen in Supplementary Figure 1**d**. Interestingly, at intermediate contour lengths ($[15, 50]\sigma$) it scales as a power-law with the same exponent (-1.17) as for rings in equilibrium. Note that the final steady-state conformations of the partly active rings are very extended and distinct from the crumpled globules found in equilibrium, as seen in Figure 2**d** and 2**e**.

Additional information on the time evolution of the hot and cold segments is provided in Supplementary Figure 2. To get more insight into the strongly subdiffusive regime of the mean squared displacements, we track the squared displacements of the centers of mass of the hot and the cold segments separately, as shown in Supplementary Figure 2**a** and 2**b**, respectively. The squared displacements of the hot segments naturally display larger fluctuations, but, more interestingly, also occasional jumps

around the plateau value. The system was simulated for $9 \times 10^6\tau \simeq 22.5\tau_{\rm diff}$ and during that time only small sudden fast relocations of the segments could be observed, after which the rings practically did not move. However, the conformations of the rings remained extended, as already seen from the mean squared internal distance profiles (Figure 2**d** and 2**e**), and also from the stable shape of the distribution of the **ch**-magnitude (Supplementary Figure 2**c**). As noted in the main text, the late-stage dynamics feature a slight decrease of the mean magnitude of **ch** in time (Figure 2**a**). We attribute such behavior to the resolution of weaker topological constraints and evolution of the system towards a locally 'deeper' steady state. In Supplementary Figure 2**d**, we show the autocorrelation function of the unit cold-hot vector $\mathbf{ch}/|\mathbf{ch}|$ for different times t_0 after the activity onset indicating a very slow decorrelation of its direction over time.

Typically, hydrodynamics plays an important role in the dynamics of active matter [1]. We neglected it here as our systems are at the melt density, for which the hydrodynamic interactions, if there is any solvent at all, are screened. Nevertheless, in fully active systems, directed flows can emerge and persist as a result of hydrodynamics with the orientational order of particles [2, 3].

Details on threadings

A ring can penetrate the surface of another ring multiple times, as indicated by the number $n_{\rm p}$. We take into account only those threadings that are longer than the entanglement length because very short ones are not model independent [4]. Note that in such case the number of the counted penetrations of a surface is not necessarily even as required by the nonconcatenation condition. We see that the mean number of penetrations per ring increases with time and reaches a plateau in the glassy phase, as shown in Supplementary Figure 3**a** and 3**b**.

We investigated whether threadings in the glassy state are responsible for the local enhancement of mechanical stress. Although momentum is injected into the present non-equilibrium system, we disregarded the kinetic contribution to the stress and concentrated only on the mechanical consequences of the system's geometrical state. To do so, we computed the Cauchy stress tensor for each particle i:

$$S_i = -\sum_{j\neq i} \mathbf{r}_{ij} \otimes \mathbf{F}_{ij} \tag{2}$$

where $\mathbf{r}_{ij}$ is the relative position of a neighboring particle j with respect to i, $\mathbf{F}_{ij}$ is the force acting between i and j and $\otimes$ denotes a dyadic product. We computed S_i for 200 consecutive time steps of the simulation in the glassy regime. Then, we divided the simulation box into sub-boxes of size 6σ and computed $-\langle Tr(S)\rangle/3V_0$ ($V_0 = (6\sigma)^3$ is the volume of a sub-box), where $\langle Tr(S)\rangle$ is the trace of the stress tensor averaged over the short sampling time and over all monomers within a given sub-box. We plotted the result as a heat map, overlaid with the locations of threadings and computed the correlation between the number of threadings and such mechanical pressure (see Supplementary Figure 3**d** and 3**e**). The resulting Pearson correlation coefficient $\chi = 0.22$ suggests a weak correlation, as, clearly, not all threadings can contribute to the pressure. A much stronger correlation ($\chi = 0.6$) is found when local density is correlated with the pressure (Supplementary Figure 3**c**).

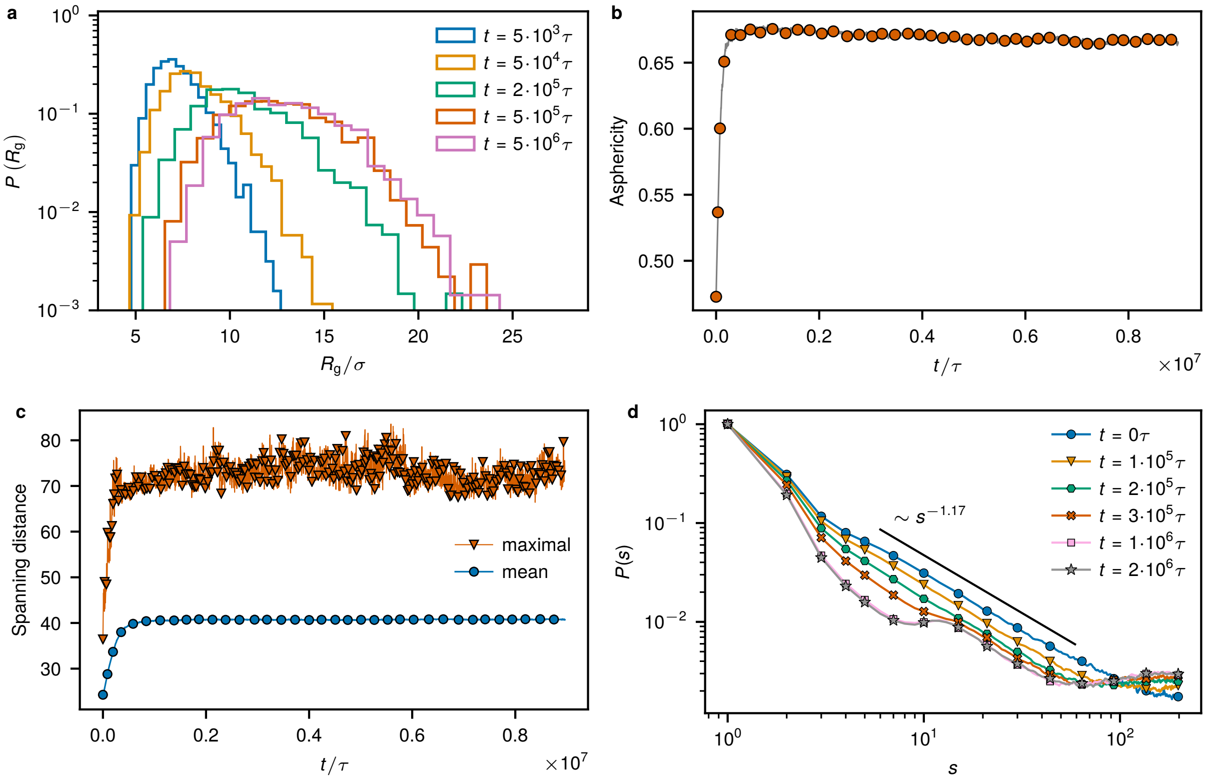

Supplementary Figure 1. **Additional shape parameters of the rings as a function of time after the onset of activity**. **a**, Time-resolved distribution of the radius of gyration. **b**, Time-resolved asphericity parameter (see Supplementary Equation (1)). **c**, Maximum and mean biggest spanning distance (in units of σ) of all rings as a function of time. At all times, all rings are smaller than the the linear box size ($L = 90.97$ for $N = 400$ and $M = 1600$). **d**, Contact probability $P(s)$ at different times t after the activity onset. Two monomers are considered as in a 3D contact if the distance between them is smaller than the cutoff distance 1.5σ. The $P(s)$ dependence is robust against changes in the cutoff distances as checked for values 1.12σ and 1.3σ (not shown).

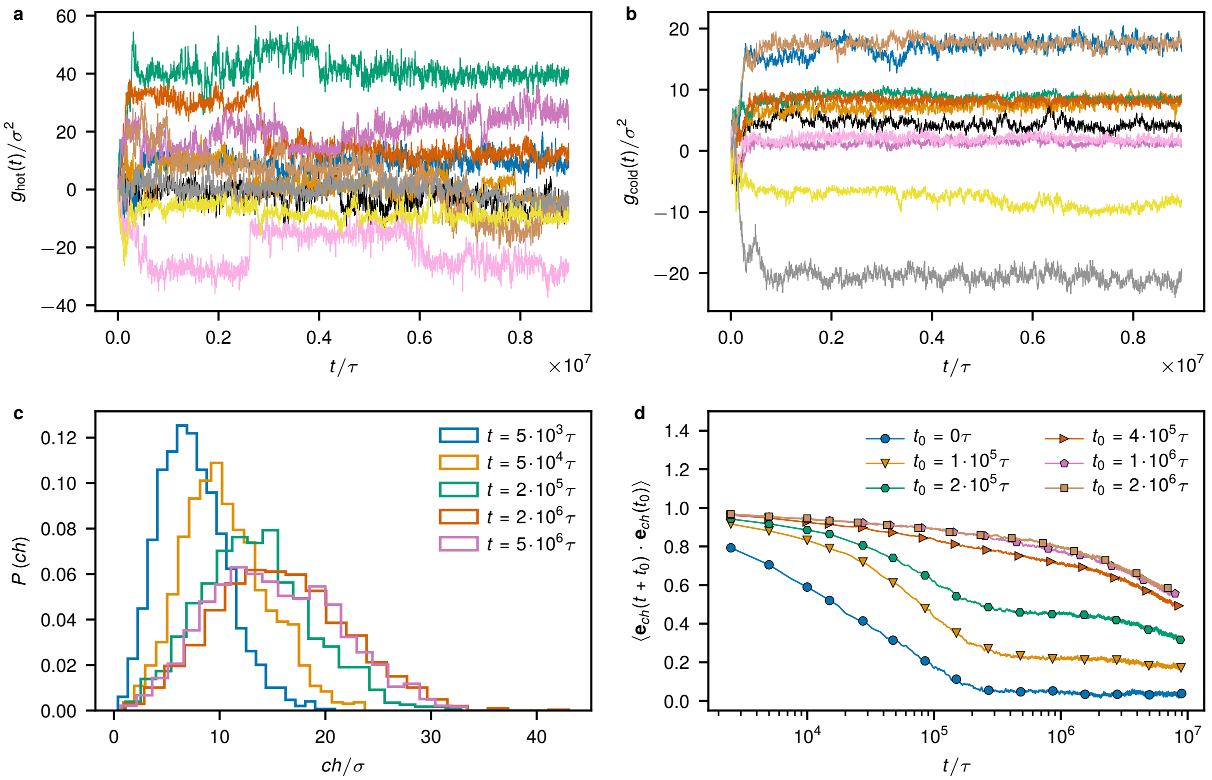

Supplementary Figure 2. **Additional details on the time evolution of the hot and cold segments**. Time evolution of the squared displacements of hot (**a**) and cold (**b**) segments of ten randomly-chosen partly active rings in the system with $N = 400$ and $M = 1600$. For clarity, we subtracted from the square displacements the initial position of each ring: $g_{\rm hot/cold} = (\mathbf{R}_{\rm hot/cold}(t) - \mathbf{C}(t))^2 - (\mathbf{R}_{\rm hot/cold}(0) - \mathbf{C}(0))^2$, where $\mathbf{C}(t)$ is the position of the center of mass of the system and $\mathbf{R}_{\rm hot/cold}(t)$ is the position of the center of mass of the cold/hot segment. **c**, Distribution of the **ch**-magnitude after different times t after the onset of activity. **d**, Autocorrelation function of the unit cold-hot vector $\mathbf{ch}/|\mathbf{ch}|$ connecting centers of mass of the cold and the hot segment of a ring for different times after the onset of activity.

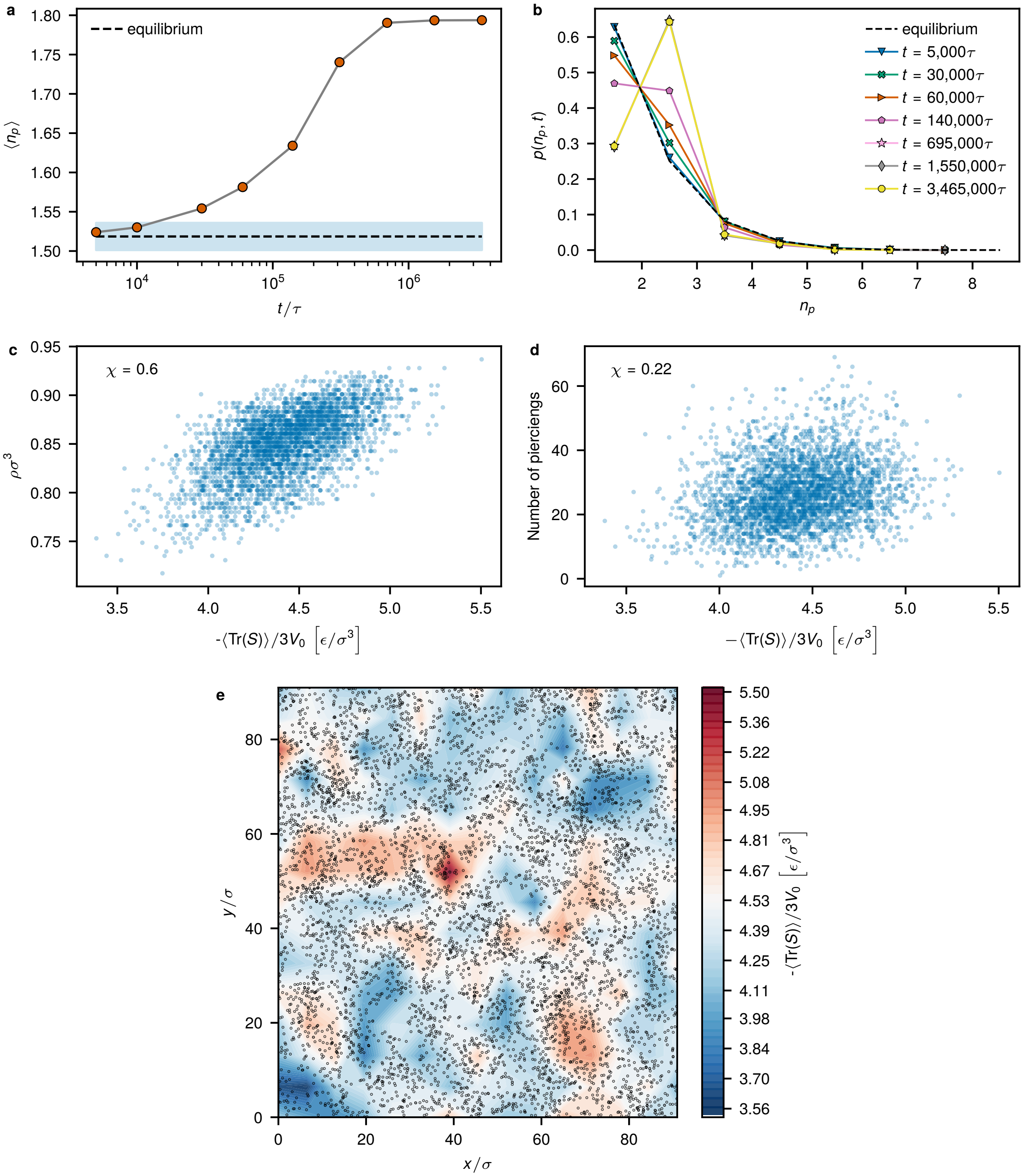

Supplementary Figure 3. **Surface penetrations statistics and correlation of threadings with mechanical stress**. **a**, Mean number of surface penetrations longer than N_e per ring as function of time from the onset of activity. **b**, Probability distribution of the number of penetrations longer than N_e per ring for different times. **c**, Correlation between the local density ρ in a given sub-box and the local diagonal stress value. **d**, Correlation between the number of threadings in a given sub-box and the local diagonal stress value. **e**, An example slab section through the simulation box. The heat map: local averaged diagonal stresses (see Supplementary Note 1 for details). Black points: locations of threadings in space.

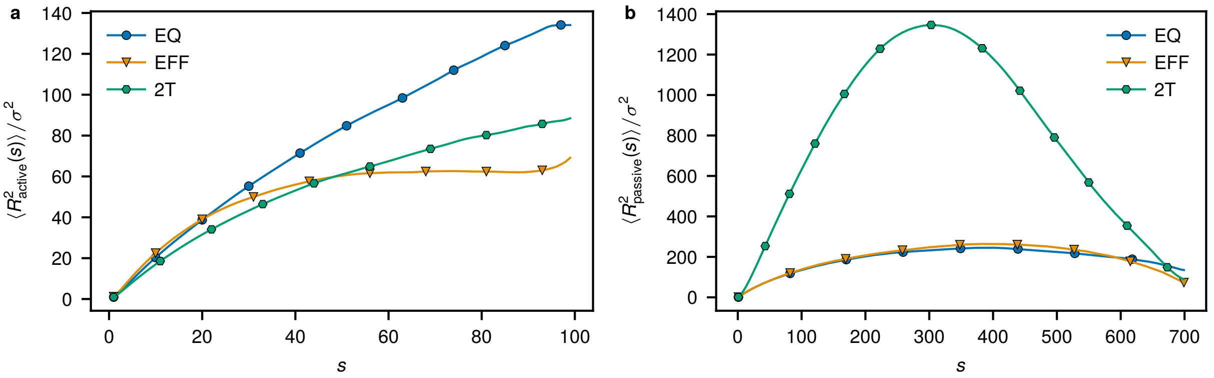

Supplementary Figure 4. **Comparison between non-equilibrium and effective equilibrium models**. The mean squared internal distance profiles of partly active rings of length $N = 800$ in a steady state in the two-temperature non-equilibrium model (2T), the segregated effective equilibrium diblock copolymer model (EFF), and the crumpled configuration of homopolymer rings in equilibrium (EQ). **a**, $\langle R^2(s)\rangle$ of the hot segment of partly active rings, or the effective model of the hot segment (see Supplementary Note 3 for details), or simply a segment of length $N_{\rm h}$ of the rings in equilibrium. **b**, $\langle R^2(s)\rangle$ for the corresponding cold segments in every model.

Supplementary Note 2: Emergent directionality of partly active polymers

Here, we use a toy model to discuss the possibility of a directed diffusion originating from different diffusivities of the hot and cold segments of a partly active polymer. We consider stochastic dynamics of two bonded particles that are coupled to two distinct thermostats (for simplicity, in 1d):

$$m\ddot{x}_1 = -\xi\dot{x}_1 - \partial_1 U + \sigma_1\eta_1 \tag{3}$$

$$m\ddot{x}_2 = -\xi\dot{x}_2 - \partial_2 U + \sigma_2\eta_2, \tag{4}$$

where $\sigma_{1/2} = \sqrt{2\xi T_{1/2}}$ with $T_1 > T_2$ ($k_{\rm B} = 1$), and $\eta_{1/2}$ are white noises satisfying

$$\langle\eta_i(t)\eta_j(t')\rangle = \delta_{ij}\delta(t-t'). \tag{5}$$

We introduce the following set of coordinates:

$$r = x_1 - x_2, \quad R = \frac{x_1 + x_2}{2}, \tag{6}$$

and assume that $U(x_1, x_2) = U(x_1 - x_2) \equiv U(r)$. Note that in this case r coincides with the cold-hot vector **ch**. The equations of motion (3) and (4) can be now rewritten in terms of the new coordinates:

$$M\ddot{R} = -\xi_R\dot{R} + \sigma_R\eta_R \tag{7}$$

$$\mu\ddot{r} = -\xi_r\dot{r} + F(r) + \sigma_r\eta_r, \tag{8}$$

where $M = 2m$, $\mu = m/2$, $\xi_R = 2\xi$, $\xi_r = \xi/2$, and $F(r) = -\partial_r U(r)$ is the force acting on the hot particle. More importantly,

$$\sigma_R^2 = 2\xi_R\overline{T}, \quad \sigma_r^2 = 2\xi_r\overline{T}, \quad \overline{T} = \frac{T_1+T_2}{2}, \tag{9}$$

and the noises η_R and η_r satisfy

$$\langle\eta_R(t)\eta_R(t')\rangle = \langle\eta_r(t)\eta_r(t')\rangle = \delta(t-t'), \tag{10}$$

$$\langle\eta_R(t)\eta_r(t')\rangle = \left(\frac{T_1-T_2}{T_1+T_2}\right)\delta(t-t'). \tag{11}$$

Provided that $T_1 \neq T_2$, the motions of R and r are coupled through correlated noises, whereas being completely independent otherwise. The equation for the center of mass is an ordinary Langevin equation and, therefore, its motion is diffusive and isotropic in the long time limit. However, at short time scales ($\simeq m/\xi$) its velocity $\dot{R}$ can be correlated to the relative (cold-hot) vector r. In particular, as follows from (11), $\langle\Delta\dot{R}(t)\Delta r(t')\rangle \sim (T_1 - T_2)$, where $\Delta\dot{R} = \dot{R}(t) - \langle\dot{R}(t)\rangle$ and $\Delta r = r(t) - \langle r(t)\rangle$. Additionally, as shown in [5] explicitly for a slightly more general toy model, a drift of the center of mass can arise only when the friction that the beads are subject to is dependent on their relative distance r, which is not part of our model. Thus, as stated in the main text, we conclude that the superdiffusion observed at intermediate times (Figure 2**c**) is explained by the effect of topological constraints, rather than by the directed diffusion.

Supplementary Note 3: Effective equilibrium model and single ring conformations

In equilibrium linear block copolymers, a colocalization of like-blocks can drive local density changes, which force conformational changes of the chains. Such changes then result in various global morphologies, e.g. vesicles or lamellae.

To show that the colocalization or phase separation of hot segments is not the main mechanism for conformational changes of the rings, we simulate a single partly

active ring in the environment of passive rings, all of the same length $N = 800$. Initially, the radius of gyration of the chain grows in time and eventually plateaus. During this stage, the chain adopts a tree-like conformation that is substantially stretched ($R_{\rm g}^2/\sigma^2 = 680 \pm 70$ obtained as an average over 100 conformations separated by $10^4\tau$), even when compared to a linear chain of length $N/2$ in a melt with similar parameters ($R_{\rm g}^2 = 180\sigma^2$ [6]). This is true also for the system with $N = 400$ for low number fractions of active chains (see below).

To support these results, we construct a passive, effective equilibrium model consisting of ring diblock copolymer rings that, as we observe, exhibit a global segregation of the two block types. As is clear from Supplementary Figure 4, despite the spatial segregation, the rings in the effective diblock model do not exhibit a substantial conformational change when compared to the homogeneous homopolymer equilibrium state. In the effective equilibrium model, we mimic the effect of the hot monomers by representing them as beads with larger exclusion volume. To do so, we keep the FENE (2) interactions the same, while in the LJ (1) interaction we use different values of the σ parameter for the hot-hot ($\sigma_{\rm hh}$), hot-cold ($\sigma_{\rm hc}$) non-bonded interactions, and additionally $\sigma_{\rm hh}^{\rm b}$ that governs the LJ interaction of the bonded hot monomers. Guided by the fact that the two-temperature density inhomogeneity is driven by the local pressure differences, we selected the values of different σ's, based on the following criteria: fitting the pressure and mean bond length of a pure hot system ($T = 3.0$, in units of ε, for all chains) sets $\sigma_{\rm hh} = 1.086\sigma$ and $\sigma_{\rm hh}^{\rm b} = 1.024\sigma$; fitting the pressure of a fifty-fifty mixture of hot and cold chains sets the value of $\sigma_{\rm ch} = 1.067\sigma$. We do not use the angular interactions as their contribution to the pressure is of the same order as the pressure fluctuations. All these fits were performed on systems of $M = 1000$ linear chains of length $N = 40$ (in the case of two-temperature systems, Langevin thermostats with friction $\gamma = (3/2)\tau^{-1}$ were used). The bonded cold monomers have the original value of the parameter σ. In the case of partly active rings, the two hot-cold pairs of monomers that are bonded have $\sigma_{\rm ch}^{\rm b} = \sigma_{\rm ch}$. To check if the effective model shares with the original two-thermostat model other properties, besides pressure and bond length, we performed the above-outlined procedure for temperatures also below 3.0 and ran the obtained effective models of equal mixtures of hot and cold linear chains. Similarly to [7], we observe phase separation of the active-like and the passive-like chains above certain threshold of the chains (temperature) asymmetry. This threshold would correspond to the temperature of the hot chains in the two-temperature system to be below 1.75, probably close to 1.5. The true two-temperature system of linear chains of length $N = 40$ phase separates around $T_{\rm h}^{\rm crit} \simeq 2.25$, for $\gamma = (3/2)\tau^{-1}$ and is only at the onset of phase separation at $T_{\rm h} = 3.0$ for $\gamma = 2/3\tau^{-1}$ [8]. Therefore, the effective equilibrium model is somewhat stronger than its non-equilibrium counterpart, but it does not feature strong conformational changes of the chains despite its stronger asymmetry between the hot-like and cold-like particles. Hence, we expect that an equilibrium model with weaker asymmetry that reproduces more closely the non-equilibrium phase separation, does not exhibit such changes either.

Let us stress that the purpose of the effective model, as described above, is not to accurately reproduce the microscopic properties of the non-equilibrium model, as it is not clear if this was possible at all [7, 9], but simply to test whether a density inhomogeneity in a system of ring block copolymers could drive substantial conformational changes. We have shown this is not the case, as seen in Supplementary Figure 4.

Supplementary Note 4: Additional properties of the steady state

The distribution of g_3 displacements for different lag times is shown in Supplementary Figure 5**a**. As mentioned in the main text, it depends only weakly on the lag time for $\Delta > 10^5\tau$. Supplementary Figure 5**b** further illustrates the behavior of the time-averaged $g_3(t, t_0, \Delta)$ for individual rings as a function of the integration time and the failure of the time average to converge to the ensemble average at longer timescales. If the system was ergodic at these time scales, the spread around the ensemble average would decrease with time.

In Supplementary Figure 6, we show the self-part of the intermediate scattering function for q-values in range $0.02 \leq q\sigma \leq 2$ for different measurement start times t_0 after the activity onset. During the early and intermediate phase, i.e. for $t_0 < 2 \cdot 10^5\tau$, we observe that the system relaxes at high wave vectors, but intermediate and small wave vectors exhibit only the first β-relaxation stage ending up at a non-vanishing value, which we define as the non-ergodicity parameter f_q (Figure 3**b**). Although our simulations are very long, for very small q's we do not observe even the β-relaxation stage. This is likely because the present system is deeply in the glassy state. For later phases with $t_0 > 10^6\tau$, we do not even reach the first relaxation for most of the wave vectors.

It is possible that one can get closer to the glass transition point if a lower number fraction of partly active chains is used. As shown below, we varied this number and observed the onset of glassy behavior, but we leave the detailed relaxation analysis for future work.

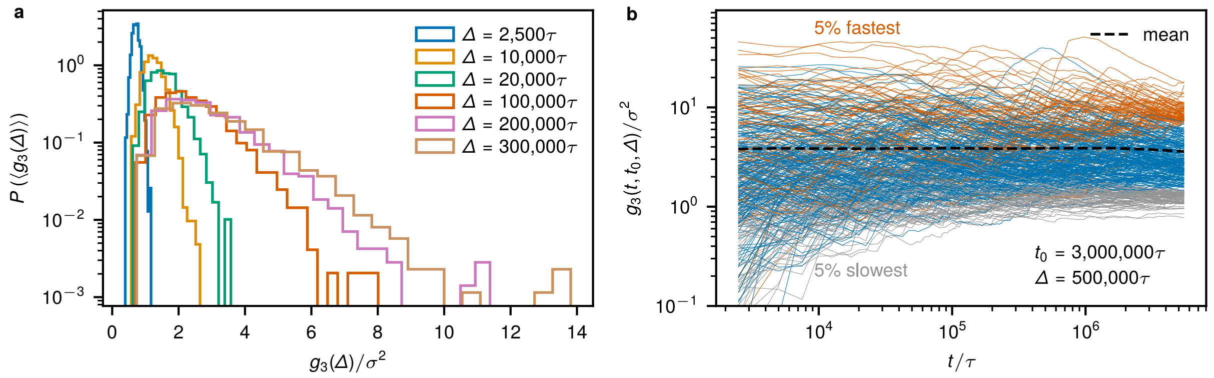

Supplementary Figure 5. **Ergodicty breaking**. **a**, Distribution of g_3 displacements for various lag times Δ. **b**, Time evolution of time averaged g_3 displacements (see Equation (8)) of individual rings as a function of the integration time t for $\Delta = 5 \cdot 10^5$ in the late simulation stage. The orange curves indicate the fastest 5% of the rings, whereas the gray ones — the slowest 5%. The dashed black line stands for the ensemble mean g_3 at a given t.

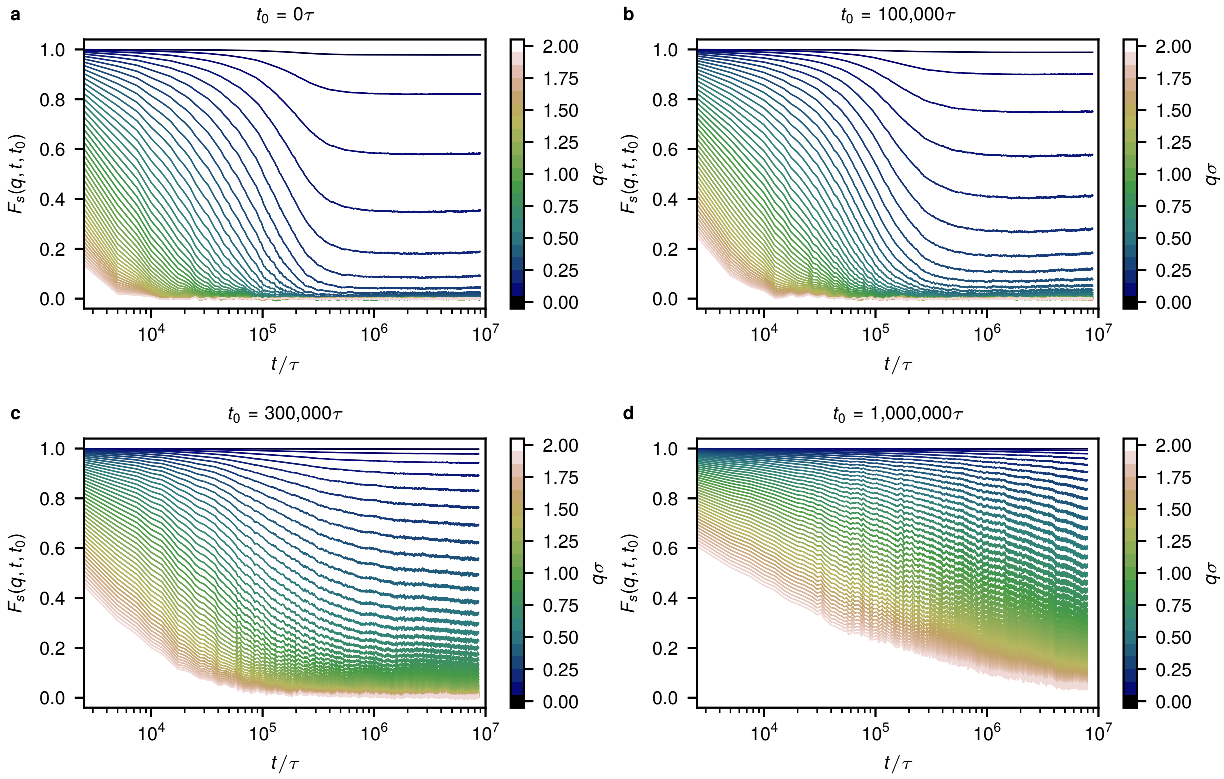

Supplementary Figure 6. **Self-part of the intermediate scattering function as a function of wave vector**. t_0 denotes the measurement start time after the activity onset. The wave vector q grow from top (blue) to bottom (orange) by step $\Delta q = 0.04\sigma^{-1}$ from the value $0.02\sigma^{-1}$.

Supplementary Note 5: Details on cut rings

The state of active topological glass is reversible. After switching off the activity of the chains, the system returns to equilibrium. We have not investigated the equilibration process in detail, but it is slower (even after $1.5 \cdot 10^6 \tau$ the system was not fully equilibrated) than the onset of the glass. This is because of the fact that the rings are purely passive and therefore their diffusivity is slower and that the mutual ring threading is higher in the glassy state and the threading is likely the bottleneck of the ring relaxation [4].

As reported in the main text, another possibility to reverse the glassiness is the elimination of topological threading constrains that are essential in maintaining the glassy state. In Supplementary Figure 7, we report the conformational properties of the rings that are in the glassy state and then cut in the middle of the cold segment. The abrupt enhancement of the system's diffusivity is shown in Figure 7**b**.

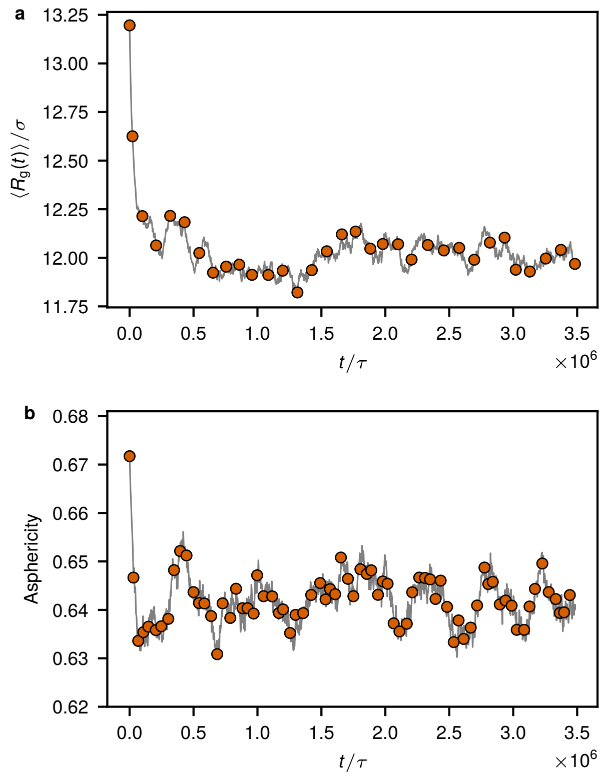

Supplementary Figure 7. **Shape properties of rings after cutting**. Mean radius of gyration (**a**) and asphericity (**b**) as a function of time after cutting happened at $t = 0$.

Supplementary Note 6: Varying number of partly active chains

We simulated systems with $M = 1600$ rings out of which only M_a were partly active while the rest were completely passive.

In Supplementary Figure 9, we plot the behavior of the radius of gyration and the asphericity for systems with different M_a separately for the active and for the passive rings. The active rings are even more stretched for lower M_a, which we attribute to the lower total number of the topological constraints as compared to system with only active rings. Based on the threading analysis, we conjecture that the active rings form more topological constraints than the passive ones due to their overall stretched configuration. If a certain number of constraints is necessary to stall the rings motion, the lower the M_a is the further each ring stretches before it reaches the number of constraints that stalls its motion. The passive rings are also slightly stretched, as compared to equilibrium, in the systems that evolve towards a glassy state.

We observe the onset of the active topological glass for M_a as low as 100, which corresponds to the number fraction $\Phi_\mathrm{a} = 0.0625$ (Supplementary Figure 9**e** and 9**f**). As is evident from these figures, both the active and the passive rings are arrested in this case. Furthermore, as shown in Supplementary Figure 9, for lower M_a the glass onset happens later in time. This is because the glassy state is formed by the mutual threadings of the active rings, which become less frequent for lower M_a. Longer simulation of systems with even smaller Φ_a might also reveal a glassy state.

The above picture is consistent with the threading cluster analysis (see Supplementary Figure 10). This reveals that the biggest cluster is supported by the active rings interconnected with the passive ones. The passive rings themselves do not form a system-spanning cluster for any value of L_cutoff. Finally, relaxation properties of the systems with varying number of partly active rings are shown in Figure 8 and Supplementary Figure 11.

Supplementary Note 7: Varying ring length

We have simulated also systems of $M = 1600$ rings of shorter lengths, namely $N = 100$ with $N_\mathrm{h} = 12$ and $N = 200$ with $N_\mathrm{h} = 25$. The system with $N = 200$ displays very similar static and dynamic properties as the system of $N = 400$ that we reported in the main text. Importantly, the number of threaded neighbors in the system with $N = 400$ is twice that of what is found in the system of $N = 200$. Therefore, we expect the number of threaded neighbors to be extensive in N also for higher N. As shown in Supplementary Figure 12, the dynamically driven shape change increases the threading

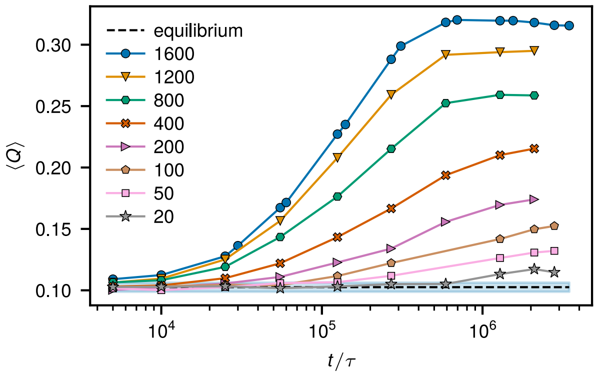

Supplementary Figure 8. Mean threading length ratio Q as a function of time for systems with different number of active rings.

and leads to a glassy state. However, the shortest system ($N = 100$) does not exhibit these properties. The main reason for this is that the hot segment is too short to drive a stronger microphase separation [7] and directional motion. Therefore, the rings do not thread excessively, and no slow-down of the diffusion could be observed. Moreover, based on the threading analysis of the glassy systems, we observe that the glass emergence is connected with the clusters of threadings deeper than $3N_e$. As the $N = 100$ rings have the length about $3N_e$, the threadings can be at most $1.5N_e$ deep and therefore the rings are too short to observe the glass.

Additionally, we have also simulated a system with longer rings of size $N = 800$, but only with $M = 200$ chains. The system exhibits a glassy state, but the chains are so extended that they can wrap around the simulation box and cause unphysical dynamics. Although a larger system of such long rings is currently out of our computational necessities, it is clear the relaxation time can only increase with respect to shorter rings, as the number of possible threadings is extensive with the ring length.

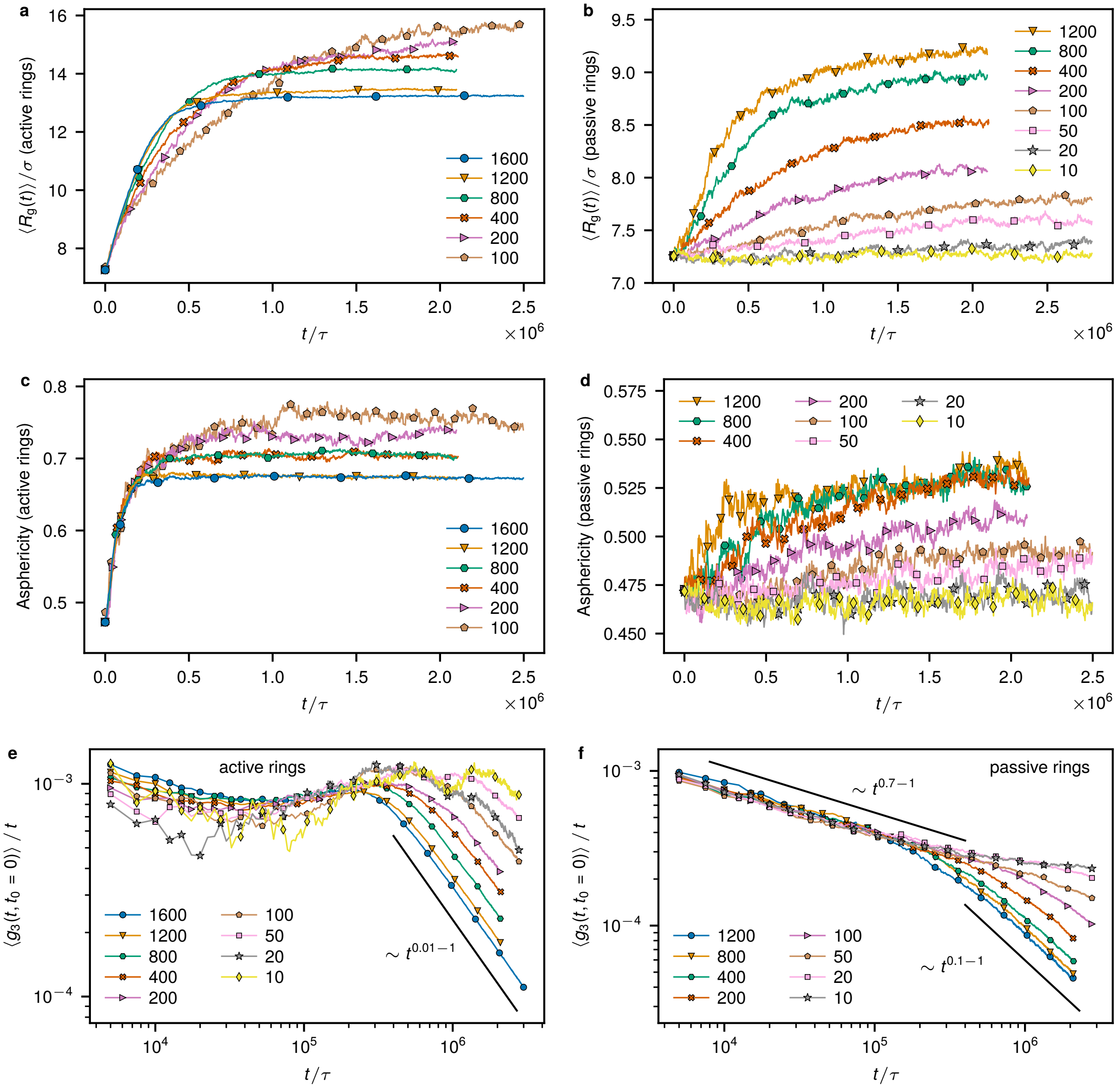

Supplementary Figure 9. **Structural and dynamical properties of rings as a function of time for systems with different number of partly active rings**. In every case, the system contains $M = 1600$ rings, out of which only $M_{\rm a}$ are active, whereas $M - M_{\rm a}$ remain passive. The rows show (from top to bottom) the mean radius of gyration, asphericity, and $\langle g_3(t, t_0 = 0)\rangle/t$ separately for active (left column) and passive (right column) subspecies in the systems with different $M_{\rm a}$, whose value is indicated in the legend of every plot.

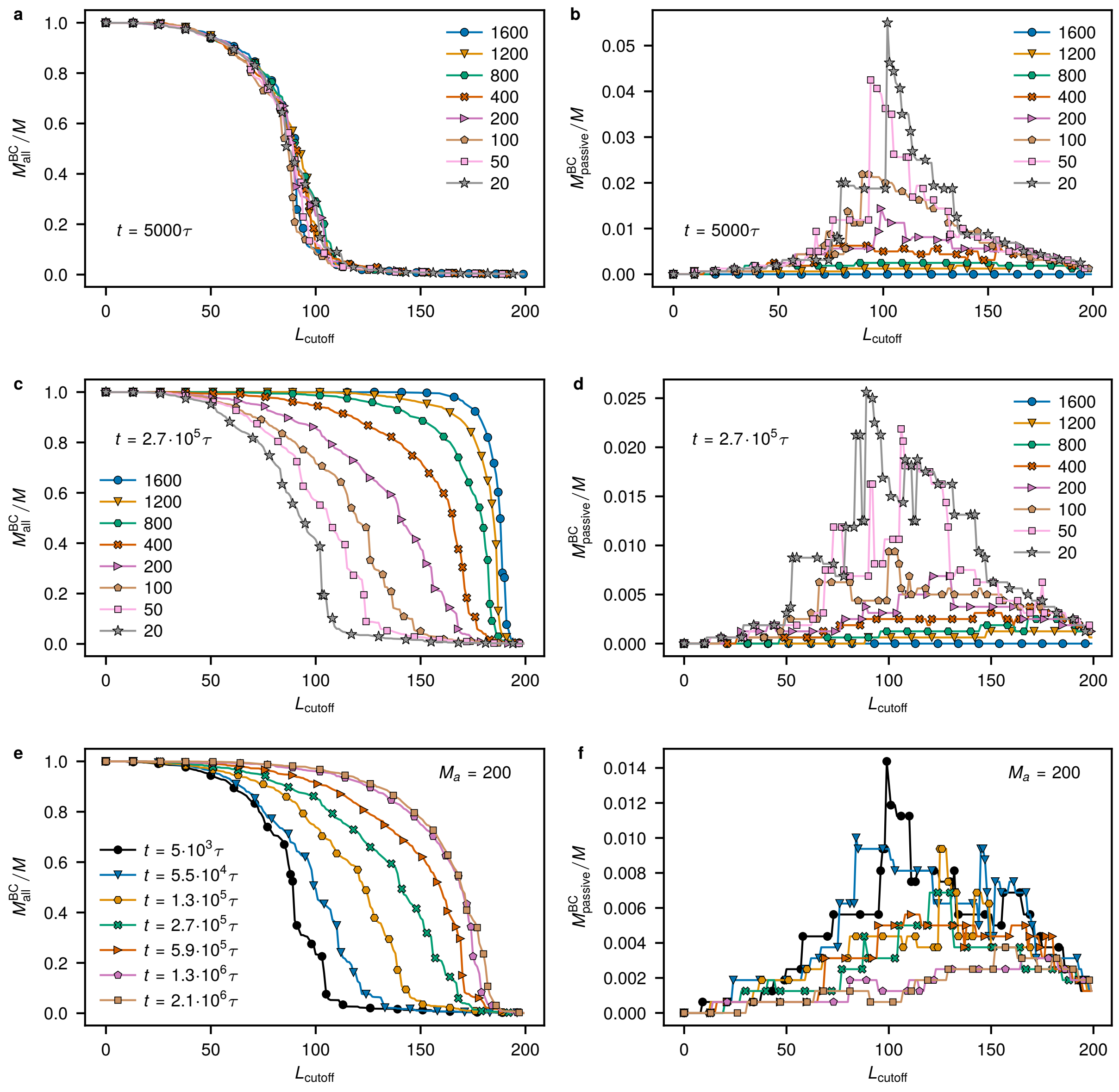

Supplementary Figure 10. **Time-resolved threading cluster analysis for systems with different number of partly active rings**. Number of rings in the biggest cluster divided by the total number of rings as a function of the cutoff length L_{cutoff} for different numbers of active rings indicated in the legend. The first row represents the state of the system at an early $t = 5000\tau$, the second one at an intermediate $t = 2.7 \cdot 10^5\tau$. The late stage is shown in Figure 8. Bottom row: time-resolved cluster sizes for the system with 200 (out of 1600) active chains. Left column: clusters that contain active (and passive) rings were used. Right column: clusters that contain only passive rings were used (see Methods). Note the difference in the scale of the ordinate for the passive ring clusters.

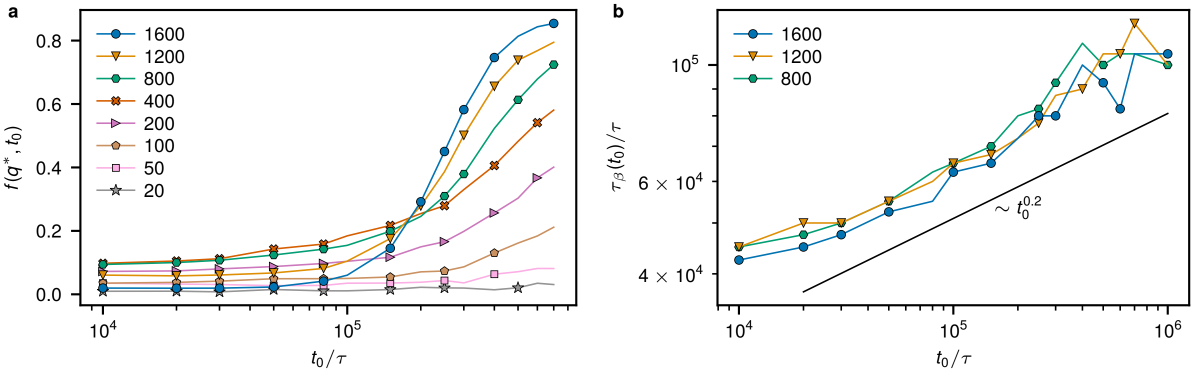

Supplementary Figure 11. **Aging of systems with different number of partly active rings**. **a**, Non-ergodicity parameter $f(q^*, t_0)$ evaluated as $f(q^*, t_0) = F_s(q^*, t, t_0)$ at $q^*\sigma = 0.35$ and $t = 10^6\tau$ to enable comparison between systems with different number of active rings that were simulated for different amounts of time as a function of the waiting time t_0 and the number of active rings in the system (indicated in the legend). The systems with lower number of active rings age 'slower', which is independently confirmed by the slower evolution of their conformational properties given in Supplementary Figure 9. **b**, β-relaxation time τ_β as a function of the waiting time t_0 for the systems with different number of active chains. For a given t_0, τ_β was computed only for the systems featuring a clear plateau in the ISF and was estimated as $\tau_\beta = (1 + F_s^*)/2$, where F_s^* is the value of the corresponding ISF at the plateau (see also Supplementary Figure 6). In all systems considered here, $\tau_\beta \sim t_0^x$ with the exponent x being close to 0.2 (black straight line).

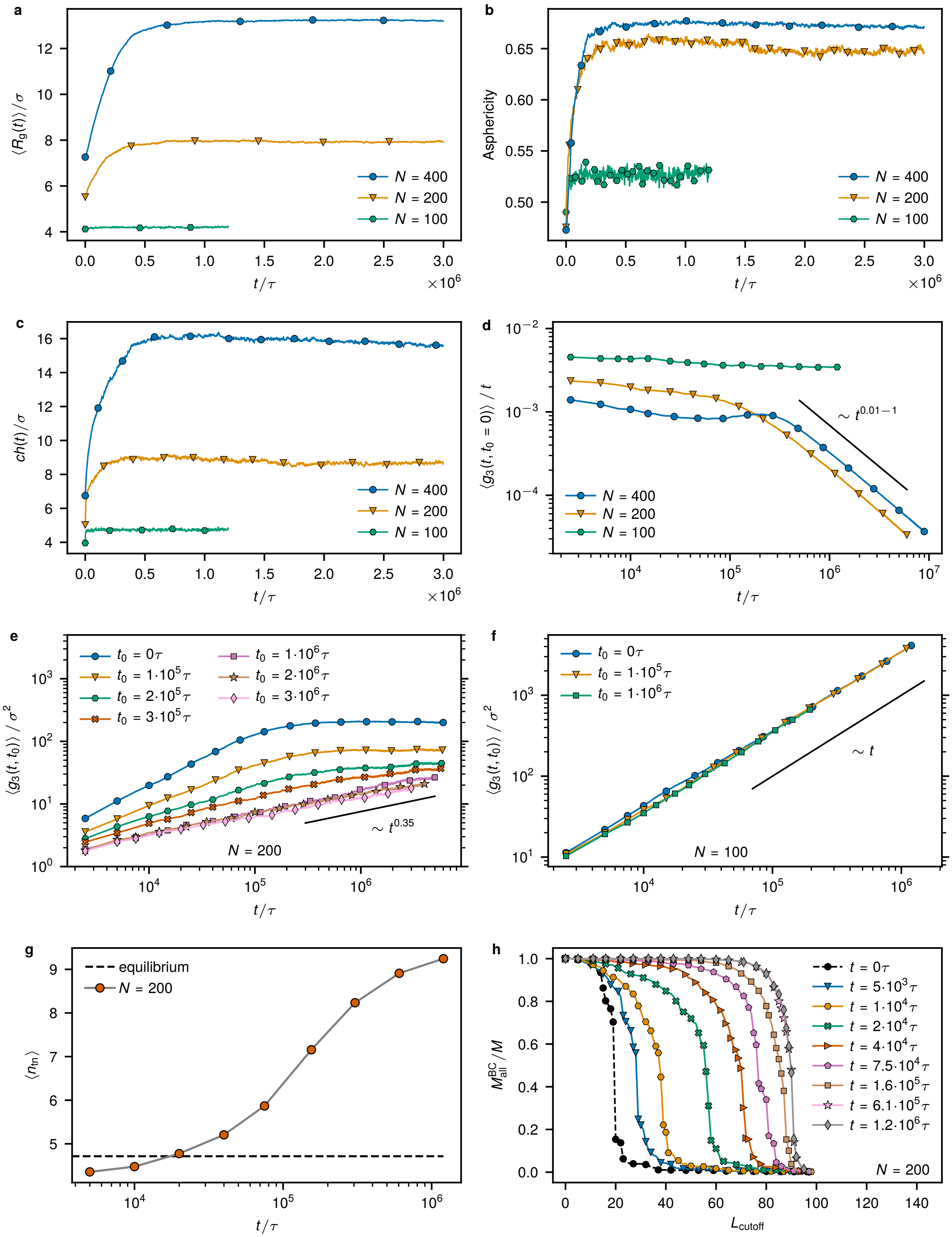

Supplementary Figure 12. **Conformational and dynamical properties of systems with rings of varying length**. The system with $N = 400$, which is extensively discussed in the main text, is included for comparison only. Time-resolved mean radius of gyration (**a**), asphericity (**b**), and the magnitude of the cold-hot vector (**c**) of the rings after the activity onset. **d**, $\langle g_3(t, t_0 = 0)\rangle/t$ for the systems of differently sized rings. **e**, $\langle g_3(t, t_0)\rangle$ for the system with $N = 200$ for different t_0 indicating the slow-down of the dynamics. **f**, $\langle g_3(t, t_0)\rangle$ for the system with $N = 100$ for different t_0 indicating fully a diffusive dynamics. **g**, Time-resolved average number of threaded neighbors for the system with $N = 200$. **h**, Time-resolved relative size of the biggest threading cluster as a function of the cutoff length L_{cutoff} for the system with $N = 200$.

Supplementary References

[1] Marchetti, M. C. *et al.* Hydrodynamics of soft active matter. *Rev. Mod. Phys.* **85**, 1143–1189 (2013).

[2] Woodhouse, F. G. & Goldstein, R. E. Spontaneous circulation of confined active suspensions. *Phys. Rev. Lett.* **109**, 168105 (2012).

[3] Sanchez, T., Chen, D. T. N., DeCamp, S. J., Heymann, M. & Dogic, Z. Spontaneous motion in hierarchically assembled active matter. *Nature* **491**, 431 (2012).

[4] Smrek, J., Kremer, K. & Rosa, A. Threading of unconcatenated ring polymers at high concentrations: double-folded vs time-equilibrated structures. *ACS Macro Lett.* **8**, 155–160 (2019).

[5] Baule, A., Kumar, K. V. & Ramaswamy, S. Exact solution of a brownian inchworm model for self-propulsion. *J. Stat. Mech.: Theory Exp.* **2008**, P11008 (2008).

[6] Halverson, J. D., Lee, W. B., Grest, G. S., Grosberg, A. Y. & Kremer, K. Molecular dynamics simulation study of nonconcatenated ring polymers in a melt. I. Statics. *J. Chem. Phys.* **134**, 204904 (2011).

[7] Smrek, J. & Kremer, K. Small activity differences drive phase separation in active-passive polymer mixtures. *Phys. Rev. Lett.* **118**, 098002 (2017).

[8] Smrek, J. & Kremer, K. Interfacial properties of active-passive polymer mixtures. *Entropy* **20** (2018).

[9] Grosberg, A. Y. & Joanny, J.-F. Nonequilibrium statistical mechanics of mixtures of particles in contact with different thermostats. *Phys. Rev. E* **92**, 032118 (2015).
