# Supplementary material for: Active topological glass
Source: Nat Commun. 2020 Jan 7;11:26. doi: 10.1038/s41467-019-13696-z (PMC6946665; doi:10.1038/s41467-019-13696-z)
Supplement: Supplementary file 3 — Description of Additional Supplementary Files [file 41467_2019_13696_MOESM3_ESM.pdf]

Description of Additional Supplementary Files.

Supplementary Movie 1 shows a time evolution of one chain of the system with $N = 400$ and $M_a = M = 1600$. The other chains are present in the system, but not shown. The video starts in the moment of the activity onset. Twenty-four frames per second are used and each frame represents a snapshot after 2500τ. The total time corresponds to $3.465 \cdot 10^6\tau$. The motion of the center of mass of the whole system was subtracted. Between 0:24 and 0:27 ($1.5 \cdot 10^6\tau$) a relatively large displacement of the hot segment can be observed, but the motion of the rings center of mass remains very limited.

Supplementary Movie 2 displays the time evolution of the threadings in the system (see main text). A colored pixel at position $(x,\, y)$ at time t signify that rings x is threading ring y at this time. The blue color means the threading is transient, while the red color indicates a persistent threading (i.e. one that survives at least until $3.4 \cdot 10^6\tau$). Only threadings among 100 rings are showed.

Supplementary Movie 3 depicts the time evolution of the chain in the system where all the rings were cut (see main text). We use the last configuration of the system in Supplementary Video 1 as the initial configuration for the present one. The video starts in the moment of cutting the rings and the total time is $2.1 \times 10^6\tau$. The chains center of mass moves significantly.
